# Insulin Production Hampered by Intermittent Hypoxia *via* Impaired Zinc Homeostasis

**DOI:** 10.1371/journal.pone.0090192

**Published:** 2014-02-25

**Authors:** Eung-Kwon Pae, Gyuyoup Kim

**Affiliations:** Department of Orthodontics and Pediatric Dentistry, School of Dentistry, University of Maryland, Baltimore, Maryland, United States of America; Cinvestav-Ipn, Mexico

## Abstract

Without zinc, pancreatic beta cells cannot either assemble insulin molecules or precipitate insulin crystals; thus, a lack of zinc concentration in the beta cells would result in a decreased insulin production. ZIP8 is one of the zinc uptake transporters involved in zinc influx into the cytosol of beta cells. Thus, if ZIP8 is down-regulated, a decreased insulin production would result. We assumed that intermittent hypoxic exposure to the beta cells may result in a decreased production of insulin due to a lack of zinc. To test this hypothesis we harvested pancreatic islets from the rats conditioned under intermittent hypoxia (IH) (fluctuating between 20.5% and 10% every 4 min for 1 h) and compared the results with those from control animals and islets. We also compared their insulin and glucose homeostasis using glucose tolerance tests (GTT) after 3 weeks. GTT results show a significant delay (P<0.05) in recovery of the blood glucose level in IH treated pups. ZIP8 expression in the beta cell membrane was down-regulated. The zinc concentration in the cell as well as insulin production was significantly decreased in the islets harvested from IH animals. However, mRNA for insulin and C-peptide/insulin protein levels in the total cell lysates remained the same as those of controls. When we treated the beta cells using siRNA mediated ZIP8, we observed the commensurate results from the IH-treated islets. We conclude that a transient IH exposure could knockdown ZIP8 transporters at mRNA as well as protein levels in the beta cells, which would decrease the level of blood insulin. However, the transcriptional activity of insulin remains the same. We conclude that the precipitation process of insulin crystal may be disturbed by a lack of zinc in the cytosol that is modulated by mainly ZIP8 after IH exposure.

## Introduction

In our previous report, we confirmed that a 5 h intermittent hypoxic (IH) challenge to neonatal pups would result in diabetogenic effects such as an increased blood glucose level and a decreased blood insulin level without any significant morphological changes in pancreatic islets [1]. Considering the pandemic incidence of diabetes and comorbid sleep disordered breathing (SDB) particularly during pregnancy [2], [3], a report showing a cause-effect relationship between IH challenge and insulin deregulation *via* a disturbed zinc homeostasis is crucial. Since recurrent IH events representing a hallmark of SDB often accompany type 2 diabetes (T2D) in the patients with SDB, a causal relationship of IH with diabetes could be assumed. Of the two kinds of zinc transporters in pancreatic beta cells, the ZIP (Zrt- and Irt-like protein from *Slc39a* genes) family takes zinc from extracellular spaces or from intracellular organelles, and transfers them into the cytosol [4], [5]. Therefore, a decreased concentration of ZIP transporters may indicate a decreased net zinc concentration in the cell. An insufficient zinc level inside the insulin manufacturing organelles such as endoplasmic reticulum (ER) or vesicles may result in hypoinsulinemia. Recently, several research groups offered that ZnT transporters (from *Slc30a* genes) transporting zinc out of the cells or organelles particularly ZnT8 as a culprit responsible for gestational diabetes [6], type 1 diabetes [7] and type 2 diabetes [8]. On the other hand, studies on the *Slc39a8* gene for ZIP8 transporter in relation to diabetes are rare. A study recently published reported the *ZIP8* gene contribution to obesity in humans [9]. ZIP8 is reported to exist in vesicles in the beta cell cytoplasm [10].

Pancreatic islets are a tissue particularly vulnerable to IH because reactive oxygen species (ROS) are produced in beta cells over the course of insulin synthesis due to disulfide bonds in proinsulin structure [11], [12]. Each disulfide bond is formed over oxidative folding in these secretary molecules which produces a single ROS in the endoplasmic reticulum. Accumulated zinc inside the islets is to counteract the enormous ROS accumulation [13], [14], [15]. Therefore, an insufficient amount of zinc in the islets could lead to apoptotic damage in the beta cells. Our previous study, however, demonstrated no change in counts or mass measurement in beta cells despite of a significant decrease in blood insulin level after IH treat, yet C-peptide production was maintained with no change [1]. We presumed that IH challenge would have resulted in no inflammatory response, but have disrupted the assembly line of insulin molecules. We hypothesize that IH challenge disrupts zinc homeostasis. We assume that, after IH challenge, C-peptides are synthesized, but insulin crystals are not matured to be precipitated due to a lack of zinc in the cytoplasm [16], [17]. We will test this hypothesis on our animal model and on the islets harvested from the IH treated animals.

## Methods

### Preparation of animals

Detailed methods have been reported in previous publication [1]. In short, near end-term pregnant Sprague-Dawley rats were maintained until parturition. On the first day of birth, dams along with their pups were randomly selected and designated the control and IH groups. The animals were housed in commercially designed chambers with food and water accessible *ad libitum*, as previously described [18]. The experimental group was maintained at oxygen concentrations that alternated between room air, 20.5% and 10% every 240s for 1 h; and the control animals were maintained in room air oxygen concentration for 1h. Number of pups per each colony was controlled the following day. The pups spent approximately 20–30 min every day with experimenter(s) to minimize stress during the time of procedures. This study was carried out in strict accordance with the recommendations in the Guide for the Care and Use of Laboratory Animals of the National Institutes of Health. The protocol was approved by the Institutional Animal Care and Use Committee of the University of Maryland, Baltimore (Permit number D121101). The entire process was conducted by the highest principles of animal welfare, and all efforts were made to minimize suffering or stress. All data were collected from male animals only.

### Glucose Tolerance Tests

Glucose tolerance tests (GTT) were performed on a separate day on two sets of control (n = 9) and experimental IH animals (n = 8) without anesthesia or sedation. The pups were separated from mothers, so deprived of food or milk 2 h prior to the test. Glucose (1.0 g/kg) was injected i.p. and blood was sampled from the tip of tails at each time point. We used 2 h protocol instead of a usual 6–8 h food deprivation since a lengthy starvation and stress in young pups could induce glycogen conversion to glucose. A glucometer and GS550 strips (Bionime, GM550) measured the level of glucose at baseline, 2, 5, 10, 15, 30 and 60 min time points as previously reported [1].

### Euthanasia and blood procurement

Pups were fasted for 2 h prior to euthanasia using CO_2_ and blood was drawn from the heart immediate after the chest was open. To prepare serum, whole blood was taken and let clot in a centrifuge tube at room temperature for 40 min. The clotted blood was centrifuged at 3,000 rpm for 15 min at 4°C and the supernatant serum was transferred into new tubes for ELISA assay.

### Rat islets isolation

Pancreas was harvested as previously described [1]. Cold collagenase solution was injected into the pancreas through the common bile duct. The removed pancreas was placed into conical tube for digestion at 37°C for 8 min in collagenase, followed by two-times washing using G-solution (1% BSA containing Hank's balanced salt solution) to dilute collagenase which slows down the digestive process. Then, the tissue was filtered through a Netwell Insert 500 µm Polyester Mesh (Corning Inc. NY). The flow-through was centrifuged at 1,000 rpm for 2 min, and the pellet was re-suspended with Histopaque 1100 solution (1077 and 1119 mixed; Sigma-Aldrich, MO) for gradient separation by centrifuging at 1,200 rpm for 20 min. The supernatant was transferred into a new tube and re-suspended and centrifuged in G-solution twice. The pellet containing islets was re-suspended in RPMI 1640 media (Mediatech Inc., VA), supplemented with 10% FBS and 1% Penicillin-Streptomycin mixture and cultured at 37°C and 5% CO_2_ incubator for 4 h to allow production of insulin and C-peptide.

### Immunofluorescence Assay

Islets were cultured in the Lab-Tek Chamber Slides (Thermo Fisher Scientific Inc., NY) which pre-coated with CELL-TAK adhesive (Becton Dickinson Bioscience, MA), for 24 h to allow proper attachment on the surface, and then fixed in CytoCell Fixative solution (Biocare Medical, CA) for 20 min. After 15 min blocking with CAS-BLOCK (Invitrogen, MD), islets were stained with anti-ZIP8 antibody and anti-pan-Cadherin (Pierce Biotechnology Inc., IL) or anti-Insulin (GenScript, NJ) antibodies at room temperature for 2 h. After washing with PBS for three times, Alexa Fluor 488- and 594-conjugated secondary antibodies (Invitrogen, OR) staining was performed for 1 h. The slides were mounted in Vectashield mounting medium with DAPI (Vector Laboratories, CA). Digital images of samples were obtained using the Zeiss LSM 510 META Confocal Microscope (Carl Zeiss, NY).

### ELISA assays for Insulin and C-peptide

Assays for secreted or produced insulin and C-peptide were performed on serum and islets. Each sample was quantified using an Insulin or C-peptide ELISA Kit (EMD Millipore Corp., MA) in accordance with manufacturer's protocol. The same amount of serum samples were incubated on the each specific monoclonal-antibody coated plate with biotinylated capture antibody for 2 h, followed by incubation with a horseradish peroxidase-conjugated streptavidin. TMB (3,3′,5,5′-tetramethylbenzidine) substrate and the stop solution were added for the reaction getting a color. Absorbance was measured at 450 nm in a spectrophotometer (BioTek Instruments Inc., VT).

Islets were collected into a tube with media and centrifuged at 500× g for 2 min. Each supernatant was taken from control and IH islets in new tubes and processed as described in our previous publication [1]. Pellets were washed with 1× phosphate-buffered saline. Each pellet was incubated with RIPA buffer (Sigma-Aldrich, Inc) containing protease inhibitor cocktail (Roche Applied Science) for 15 min on ice to extract whole cell lysate, and centrifuged at 13,000 rpm for 15 min. 10 µg of cell lysate was used to estimate the amount of insulin and C-peptide produced.

### Western Blot Assays

Collected islets were prepared for whole cell lysate as previously prepared for ELISA assays. Cytosolic and plasma membrane fractions were prepared using a subcellular protein fractionation kit (Pierce Biotechnology Inc., IL). Thirty µg of proteins were resolved on the SDS-PAGE and transferred onto a PVDF membrane using an electroblotting method. After blocking with 5% milk TBS-T, the membrane was stained with primary antibodies (anti-ZIP8 and anti-pan-Cadherin antibodies, Pierce Biotechnology; anti-β-actin antibody from Cell Signaling Technology) followed by a horseradish peroxidase-conjugated secondary antibody. Chemiluminescent detection reagents (Pierce Biotechnology Inc., IL) were used to detect immunoreactive proteins and exposed to X-ray films. Density measurements were carried out by Multi Gauge v3.0 (Fujifilm, Japan), and relative values were calculated on the subtracted quantities between ZIP8 and β-actin bands.

### Zinc assay

Levels of zinc in the whole cells were quantified using Zinc Colorimetric Assay Kit (BioVision Inc. CA) in accordance with the manufacturer's protocol. Thirty μg of cell lysate was de-proteinized by adding 7% TCA solution, and centrifuged for precipitation. The supernatant was mixed with the zinc reagent in the 96 well-plate. Absorbance was estimated at 560 nm in Epoch spectrophotometer (BioTek Instruments Inc. VT).

### RNA Interference

Harvested islets were infected with recombinant lentiviral particles containing short interfering RNA (siRNA) for the rat *Slc39a8* (*ZIP8*; Accession Number NM_001011952.1) gene or scrambled siRNA (Applied Biological Materials Inc., B.C., Canada) for three days. The target sequence are: *Slc39a8*-393, TGG ATT CTT GTC AGT GAC AAT CAT CAA TT; *Slc39a8*-537, CCA GCT TAT TCC AGA GGC ATT TGG ATT TA; *Slc39a8*-890, CCA AAC TGT CAG AAA TAG GAA CGA TTG CT; *Slc39a8*-1290, GGA CTT CAC CTT CTT CAT GAT CCA GAA CG. Packaging lentivirus was carried out on the Lenti-X 293T cell (Clontech Laboratory Inc., CA) with the second Generation Packaging Mix (Applied Biological Materials Inc., B.C., Canada) by transfection with X-tremeGENE HP DNA Transfection Reagent (Roche Diagnostics, IN). The culture media containing lentiviral particles was concentrated with Lenti-X Concentrator (Clontech Laboratory Inc., CA) in accordance with the manufacturer's protocol.

### Quantitative RT-PCR

Total RNAs were purified using the RNeasy Mini Kit (QIAGEN Sciences, MD) on harvested islets. First-strand cDNA was synthesized from 2 µg of RNAs using the High Capacity cDNA Reverse Transcription Kits (Applied Biosystems, CA) primed with a mixture of random primers. With the mixture of 25 µl volume of 1× SYBR green master solution (Applied Biosystems, CA) containing 2 µl of cDNA template with 5 pmol of primers on the 96 well real-time PCR plate (Eppendorf, Germany), quantitative PCR was performed with the Eppendorf realplex system (Eppendorf, Germany). Amplification was triplicated for each sample. Each primer set was designed like the following; *Slc39a8*-Forward (AAA CGT CAC CCA GAT AAC CAG), *Slc39a8*-Reverse (GAC AAG AAT CCA TAG CCC CAG), *Ins1*-Forward (ATC TTC AGA CCT TGG CAC TG), *Ins1*-Reverse (GGC TTT ATT CAT TGC AGA GGG), *Gapdh*-Forward (CCA TCA ACG ACC CCT TCA TT), and *Gapdh*-Reverse (GAC CAG CTT CCC ATT CTC AG).

The threshold cycle (C_T_) for each reaction was determined as quantity of gene expression. The difference in average C_T_ value between *Gapdh* housekeeping gene (Accession Number NM_017008.4) and the target genes (*Slc39a8*, Accession Number NM_001011952.1 and *Ins1* gene, Accession Number NM_019129.3) was calculated and log-transformed for each sample to be termed into ΔC_T_ values. The value of ΔC_T_ was further normalized to show relative expression levels with respect to the mean value.

### Statistics

For point-to-point comparisons of glucose levels between control and IH groups at each time-point, we used two-tailed t-tests (n = 9 for control and n = 8 for IH). For group comparisons of the insulin and C-peptide harvested from the same numbers of pups, two-tailed t-tests were performed. Each assay was repeated at least three times. On the results of ELISA, t-tests were used to assess differences between the groups. The samples for ELISA assay were pooled from two animals.

## Results

**Figure 1:**
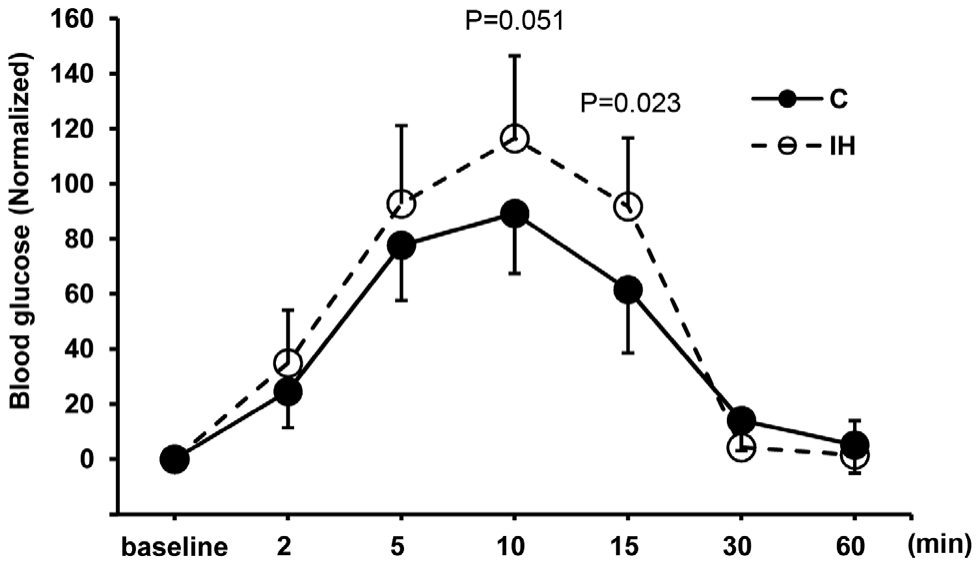
Glucose tolerance tests (GTTs). GTT results of 1(C) and IH treated (IH) animals at 15 min point (P<0.05). At 30 min point, the glucose level returns to the baseline in both control and IH treated groups.

**Figure 2:**
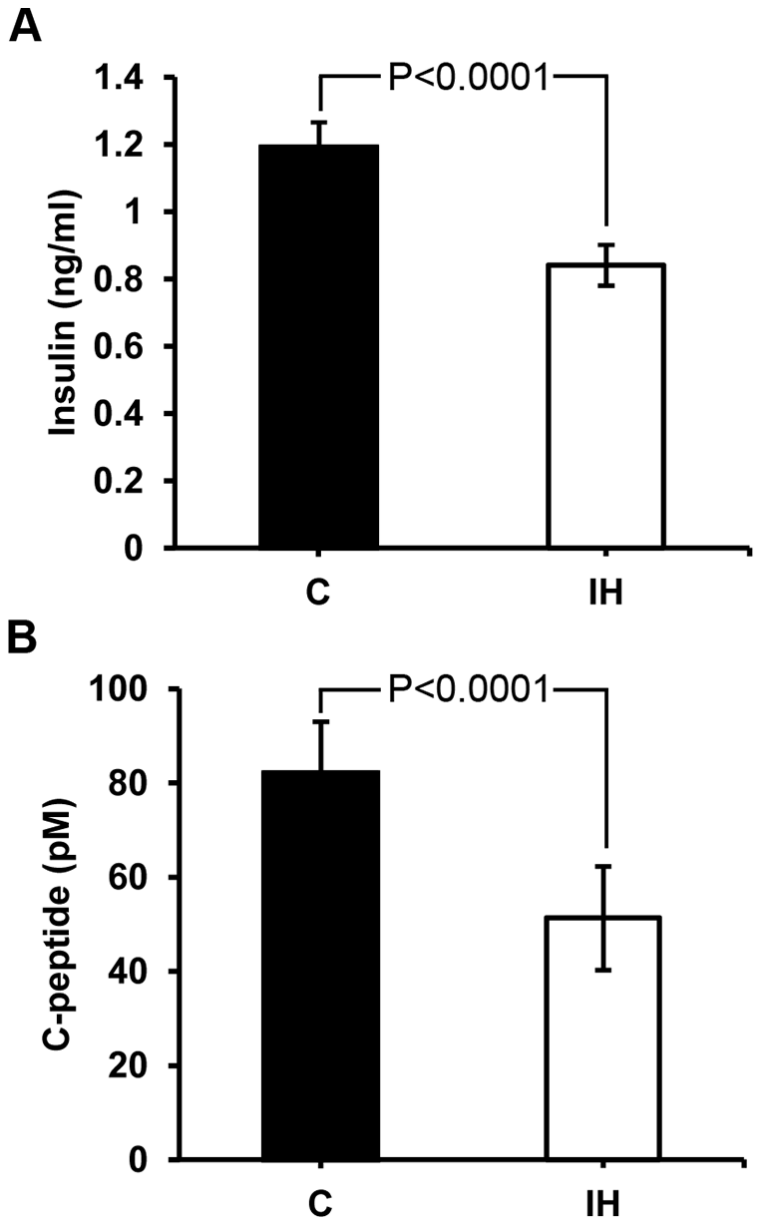
Serum levels of insulin (A) and C-peptide (B). Both insulin and C-peptide levels in serum of the treated animals are significantly lower than those of controls.

Results are presented in means ± standard deviations (s.d.) or standard errors (s.e.). All vertical bars in the graphs of figures indicate standard errors. Two groups of pups were compared in weight (42.8±6.29 g for controls vs. 50.8±2.35 g for IH, P = 0.004, where means ± s.d.). Because the IH treated pups are significantly heavier, we attempted standardizing blood glucose levels by placing all baseline measurements at 0 and converted other measurements with respect to the baseline (Fig. 1). Figure 1 shows that the blood level differs at the 15 min point at P<0.05. This result indicates that the average glucose level of 1 h IH treated pups sustains high for a longer time compared to the control pups. Before standardization, the blood glucose levels were statistically higher at 5, 10 (peak) and 15 min points in the IH pups (See Fig. S1 submitted as a supplement figure). When insulin and C-peptide levels in serum were compared, the IH group showed a significantly lower level at P<0.0001 (Fig. 2). The level of blood insulin decreased approximately 29% and the level of C-peptide decreased approximately 37.6% after IH challenge compared to the controls. Blood glucose levels and insulin levels appear commensurate.

**Figure 3:**
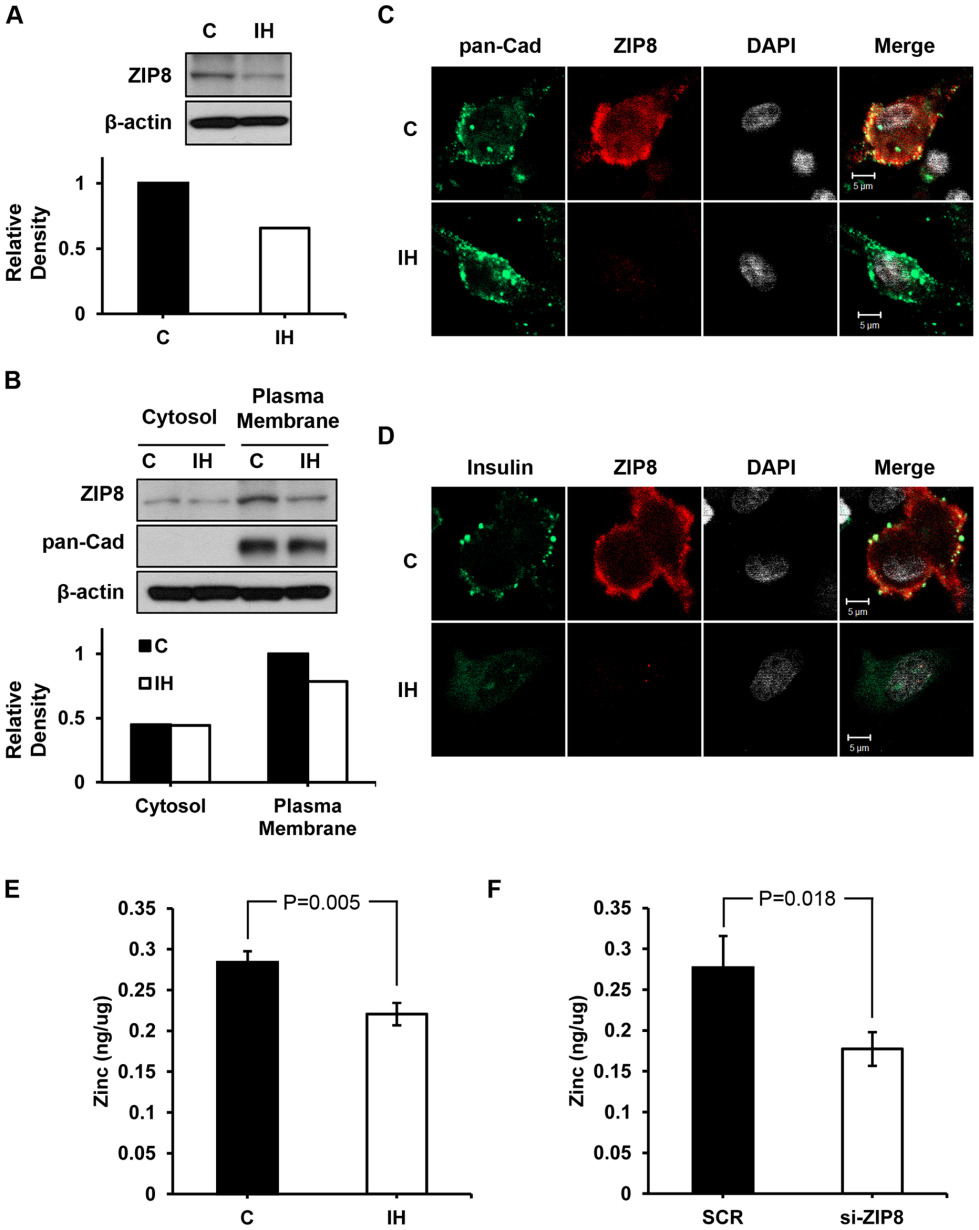
Disturbed zinc and ZIP8 homeostasis. (A) Western blot assay results on the whole cells harvested from both control and IH animals. ZIP8 protein concentration shows a noted decrease in the IH challenged group. When fractionated into cytoplasm and plasma membrane portions, (B) ZIP8 protein is expressed in the plasma membrane more than the cytoplasm portion. The IH group shows a lower density of ZIP8 in both cytoplasm and membrane. (C) Confocal images on beta cells demonstrate that ZIP8 transporters along the plasma membrane (indicated by pan-Cadherine protein resides in the membrane) markedly decreased after IH challenge. The bottom panel (D) shows that insulin expression also significantly decreased in the IH group. Scale bars indicate 5 µm. (E and F) Zinc taken up by the beta cells decreased in the IH group, yet not as much as the amount decreased in the siZIP8 treated cells (F). SCR indicates scrambled.

Since we hypothesized that IH challenge would disrupt zinc homeostasis, we examined zinc uptake transporters, particularly ZIP8 in the beta cells. Western blot assays show a lower ZIP8 expression particularly in the partitioned membrane obtained from IH treated pups than controls (Fig. 3A and B). Figure 3C depicts a significantly decreased density of ZIP8 protein 3 weeks after 1h IH exposure at postnatal day 1. The level of insulin decreased significantly as the level of ZIP8 transporters decreased in the IH group (Fig. 3D). As the expression of ZIP8 zinc uptake transporters in the plasma membrane decreased, the level of zinc (Fig. 3E) imported by ZIP8 (Fig. 3F) transporters into the cell decreased significantly after 1h IH challenge.

**Figure 4:**
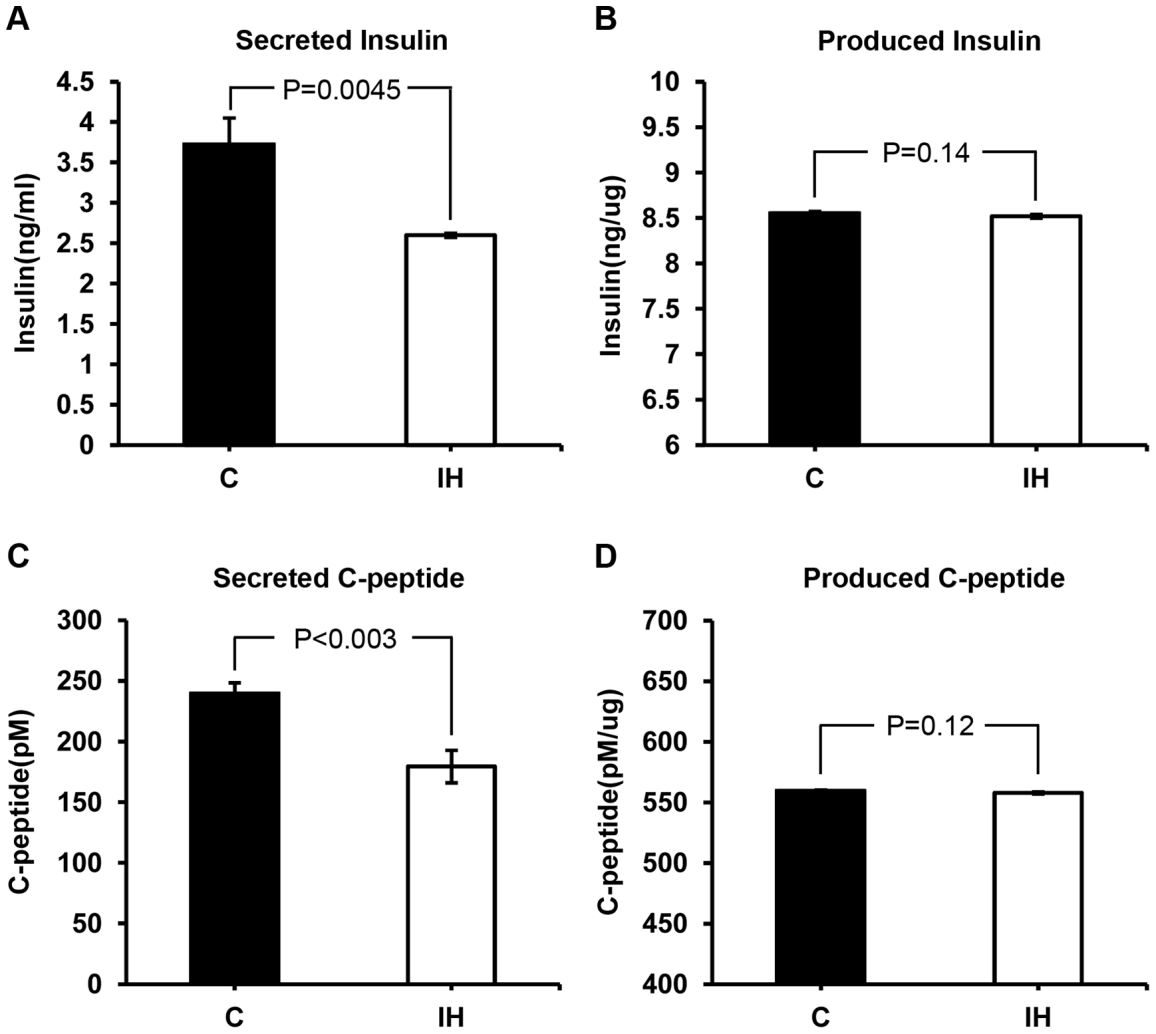
Comparisons on insulin and C-peptide concentrations between secreted amounts vs. produced amounts. The produced insulin (B) and C-peptide (D) measured in whole cell lysates do not show a difference; however, the secreted amounts obtained from each medium are significantly lower in the IH group when compared to control (A and C).

To test whether a lack of insulin in the blood is due to a lack of production, we compared the level of insulin secreted into medium and the level of insulin in the lysates harvested from the islets of IH treated and control animals. Insulin and C-peptide levels measured in the cell lysates did not differ between IH and control groups (Fig. 4B and 4D). However, the levels of insulin and C-peptide measured in the media were significantly different (See Fig. 4A and 4C). Islets harvested from IH treated pups secreted a significantly lower level of insulin (30% lower than control) and C-peptide (24% lower than control). These results and the results obtained from blood serum in Figure 2 accorded well.

**Figure 5:**
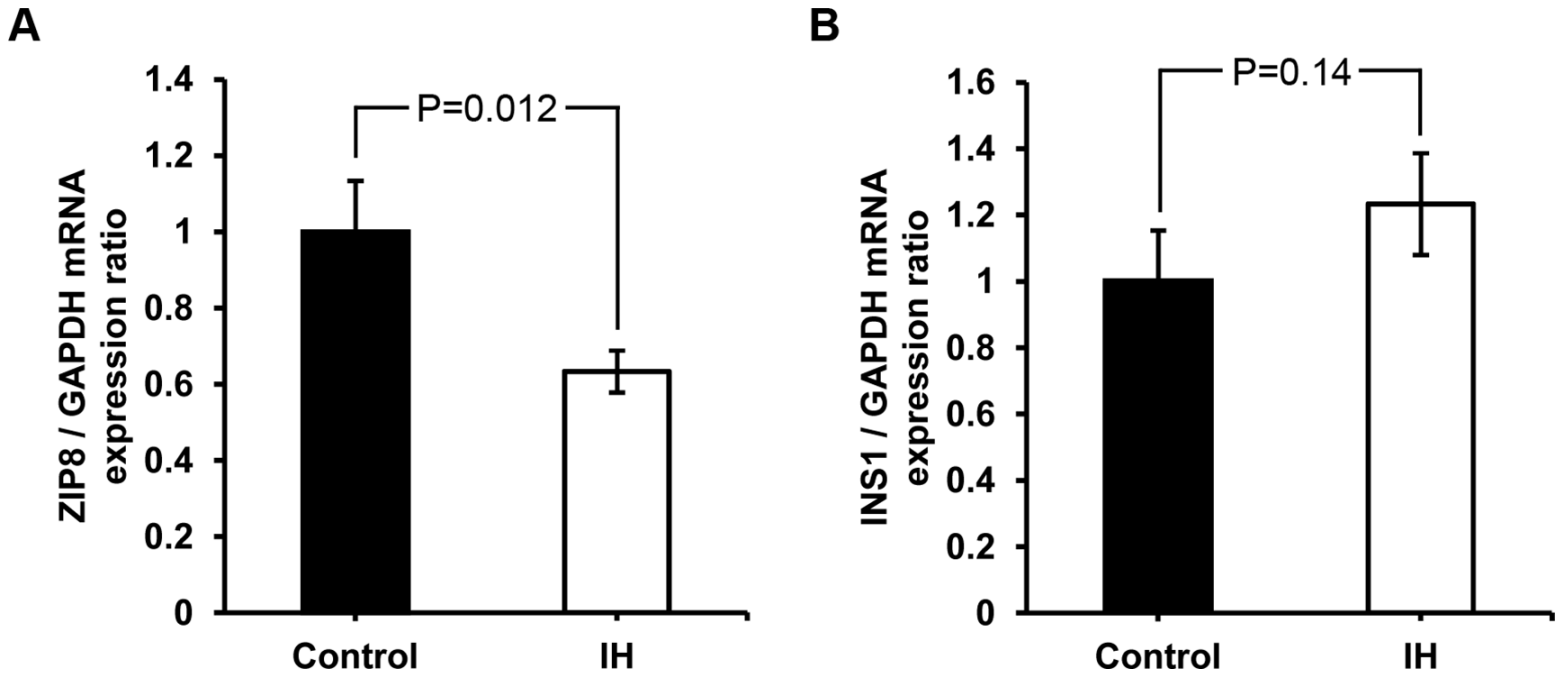
mRNA levels of *ZIP8* and *Ins1*. The mRNA level of *ZIP8* in the beta cells obtained from IH animals significantly lower than that of controls (A). The level of mRNA of the insulin gene *Ins1* remains the same (B).

Finally, we tested whether the lack of insulin secretion is due to a lower mRNA level of the *Ins* gene or *ZIP8* gene. Our quantitative RT-PCR results show that mRNA production for *ZIP8* significanlty decreased approximately 40% (at P<0.05) in the IH pups, whereas that of insulin shows an insignificant increase (See Fig. 5). This result reveals that the level of insulin mRNA concentration does accord to the level of produced insulin protein shown in Figure 4B, yet does not comensurate either with the blood insulin level in Figure 2A or with the secreted insulin level in Figure 4A probably due to a lack of zinc, an essential element for crystalization and secretion of insulin. Zinc involves in the post-translation and secretion processes of insulin not in the transcription process.

## Discussion

### Validity of our 1 h model

Unlike our previous studies, we treated animals in IH condition for 1 h instead of 5 h. The purpose of decreasing the treatment time was two folds: first, 1 h (or 8 times) of mild IH episodes is more clinically relevant than 5 h regimen. Eight times of reoxygenation after 7 times of mild hypoxic events is a clinically realistic conditioning model that would be more often occurring in human neonates born preterm who are commonly vulnerable to hypoxic events [19], [20]. Secondly, our previous study showed occasional proinflammatory cytokine expression in the islet tissue after 5 h IH treatment. We wanted to minimize a potential cytokine involvement by decreasing our IH challenge regimen to 1 h.

Our 1 h IH treated animals still revealed a significant difference at 15 min point on the GTT curve when compared to that of controls. Because the predicted difference was assumed to be small compared to the result we previously obtained from 5 h model, we added multiple colonies of pups and adjusted the curve with respect to each baseline measurement. The slight but significantly delayed recovery from hyperglycemia observed in the IH group may indicate that the beta cells after IH treatment become dysfunctional (Fig. 1), and that is why the levels of insulin in the IH animals are low (Fig. 2).

### Role of zinc in insulin production

Disturbance of zinc homeostasis has been accepted as one of the most crucial signs of diabetes because diabetic patients experience an increased urinary excretion of zinc [21] and zinc is involved in the synthesis, secretion as well as function of insulin. The zinc level in serum as well as in the cytoplasm of beta cells in diabetic patients is low compared to normal levels irrespective of types of diabetes [22]. Despite the general congruence in opinions, some clinical study defies a one-to-one relationship between the zinc level in beta cells and the level of insulin secretion. Our current results show that insulin production decreases as the intracellular zinc level decreases after IH challenge; however, mRNA production of insulin does not show any change. This result may indicate IH insults do not influence the beta cells at the transcription level of insulin, but may influence the assembly process in the production line.

The human body contains 2–4 g of zinc in total, but the mobile amount of zinc in plasma is very small, i.e. approximately 12–16 µM [23]. Mainly, zinc is bound to proteins such as metallothionein in cytoplasmic compartments of zinc abundant cells like pancreatic beta cells. Zinc accumulated in the ER and Golgi apparatus in pancreatic beta cells plays a dual function, i.e. anti-oxidative function [24] and insulin production/secretion function [25]. This may be why our IH model is extremely prone to be diabetic. The additional ROS accumulation from our IH challenge should have consumed zinc in the insulin containing vesicles, ER, as well as Golgi apparatus.

Some recent studies view zinc as an intracellular second messenger augmenting insulin activity *via* ZIP which plays a role of sensor [26], [27]. In fact, there is ample evidence showing that increased zinc levels can boost insulin production and secretion. Hence, Myers *et al.* [26] explained that the increased zinc concentration in the cytoplasm would increase numbers of ZIPs as ZIP7 did in another study [28], then as a result, increase insulin secretion. They also quoted that this is why oral administration of zinc improves symptoms in type 1 and type 2 diabetic patients [29]. However, the anti-diabetic effect of zinc is still controversial due to a weak dose-effect relationship clinically [30]. Since most zinc is bound with ligands in beta cells, whether the signaling process of zinc to alarm the innate defense system in response to a physiologic challenge by immediate ‘buffering and muffling’ is sufficiently effective remains to be answered [31], [32]. We have not performed a rescue experiment yet; however, it would be very interesting to observe whether zinc administration would recover ZIP8 expression, and in turn recover insulin secretion in our animal model as well as *in vitro* model. In fact, no reports are available as to whether zinc administration increases ZIP8 concentration in beta cells.

### Role of ZIP8 transporters in insulin production and secretion

Currently the field's understanding on the action mechanism of ZIP8 zinc uptake transporters in the beta cell is minimal. We observed that ZIP8 zinc uptake transporter, which is abundant in the normal beta cells, was down regulated in the beta cells obtained from IH exposed animals as well as in the ZIP8 down-regulated islets by siRNA mediation. This tentatively confirms that ZIP8 is a functional transporter for zinc uptake as suggested in our results and for accumulation in normal beta cells. Down regulation of ZIP8 along with the loss of zinc was associated with a beta cell insulin production despite the fact that high zinc accumulation is a typical characteristic of normal beta cells. A recent study on ZIP8 zinc uptake transporters reported that an elevated glucose level increased free cytosolic Zn^2+^ in rodent pancreatic beta cells as *Slc39a6*, *Slc39a7* and *Slc39a8* were increased [33]. However, they observed no change in these zinc importers when they elevated the level of intra- and extracellular Zn^2+^. Thus, they downplayed the role of *Slc39a8* in the pathogenesis of early diabetic phase which could be the case in mouse cells. To our knowledge, however, the current study is the first exhibition of ZIP8 zinc uptake transporters existing in the plasma membrane of rodent beta cells and a unique demonstration of a possible association of ZIP8 in relation to pancreatic islets and reduced insulin production after hypoxic challenge. This paper demonstrates the implication of zinc and ZIP8 in beta cell production of insulin and the IH treatment effects in primary beta cells.

Although productivity of insulin and C-peptide were decreased after a transient IH challenge, mRNA concentrations of insulin and C-peptide proteins maintained in a normal range. We also confirmed that the production level of C-peptide was maintained as well. This may indicate that the amount of synthesized and assembled proinsulin did not change despite the IH challenge. However, a lack of zinc in the ER and Golgi apparatus prevented the insulin molecules from being precipitated and crystalized. Therefore, the levels of secreted insulin and C-peptide in the growth medium decreased significantly, as well as in the blood.

### Potential mechanism on how ZIP8 concentration is reduced

The mRNA for *Slc39a8* gene encodes protein ZIP8 that was found in the plasma membrane and the cytoplasm of the beta cell. ZIP8 is known to import zinc at the onset of inflammation by inhibiting NF-κB probably for host defense as a part of innate immune function [14]. ZIP8 is also involved in testis damage by the toxic cadmium [34], which is often found in cigarette smoke [35]. Zinc appears to be an essential metal to fight off cadmium toxicity [36]. In this study, estimated amount of mRNA for *Slc39a8* gene was decreased in correlation to the decrease of ZIP8 protein. However, as we discussed in our previous publication, we did not observe significant corresponding proinflammatory changes even after 5 h challenge. One explanation on the down-regulated *Slc39a8* transcription activity and in turn ZIP8 production may be due to a potentially increased micro-RNA activity. In fact, a recent study reported a cause-effect association between *micro-RNA488* (or *miR488*) and *ZIP8* in osteoarthritis by involving in a reduced degradation of chondrocytes [37]. Roles of zinc uptake transporters such as ZIP8 in bone and *miR488* may be crucial since a low bone mineral density is a typical symptom of T1D and T2D [38]. We have not confirmed this epigenetic effect of IH *via* assessing amount of *miR488* in our model yet.

One may ask what would occur to zinc level if blood glucose level decreases in a typical T2D patient. A high blood glucose level despite the increased insulin level tends to maintain the level of cytokines high, and which would consume more anti-oxidants including zinc [12], [13]. Therefore, a sudden decrease in blood glucose level would increase the level of zinc shortly; however, such a tight regulatory margin of zinc in human body will bring the level to a homeostasis quickly.

Although we previously called this set of diabetic symptoms ‘perinatal diabetes’ in our recent publication, we still do not know pathophysiologic characteristics of this form of diabetes. Not only gestational IH challenge but also early postnatal IH challenge results in long-lasting alterations in the neural control of breathing [39]. Thus, effects of this type of disruption on glucose-insulin homeostasis may also progress over years or even for life. Numerous reports suggest a cause-effect relationship between SDB during pregnancy and metabolic disease in later stage of life [40]. Our results and other works set forth may provide a potential mechanism that demonstrates this cause-effect relationship.

## Conclusions

We report that a transient IH challenge to neonatal rodents could result in diabetic-like symptoms in later time of life *via* a disrupted zinc homeostasis due to a decreased concentration of ZIP8 zinc uptake transporters. The IH challenge appears irrelevant to the transcriptional process of insulin. We think that insulin production by beta cells depends on the level of ZIP8 transporter expression in the plasma membrane.

## Supporting Information

Figure S1 **GTT results from the raw measurements.** IH group shows significantly high glucose levels at the 5, 10 and 15 min points.
(TIF)
