# Supplementary figures and images for: Insulin Production Hampered by Intermittent Hypoxia via Impaired Zinc Homeostasis
Source: PLoS One. 2014 Feb 25;9(2):e90192. doi: 10.1371/journal.pone.0090192 (PMC3934988; doi:10.1371/journal.pone.0090192)

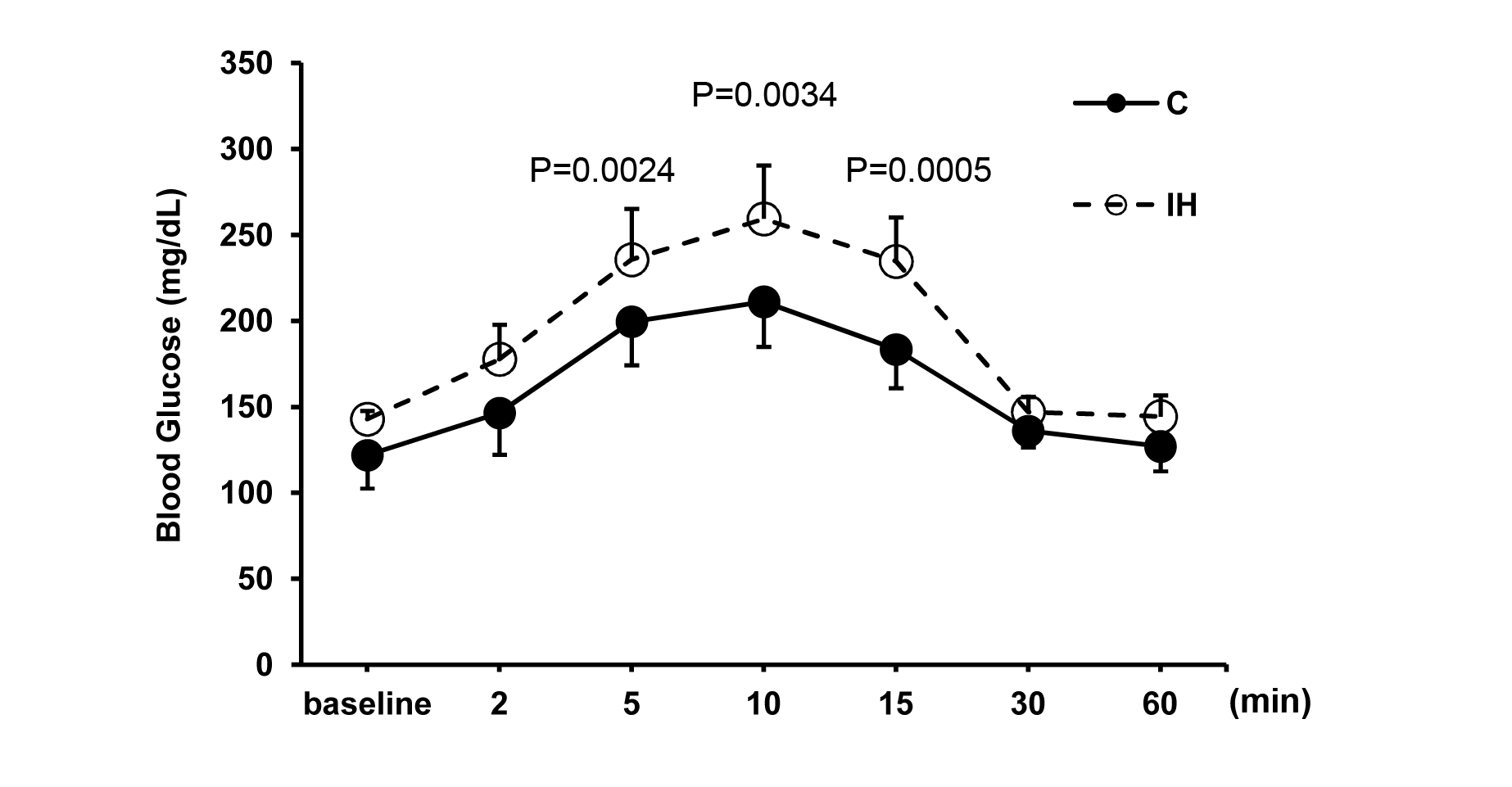

Supplement: Figure S1 — GTT results from the raw measurements. IH group shows significantly high glucose levels at the 5, 10 and 15 min points. (TIF) [file pone.0090192.s001.tif]
